# A rare case of fundal intramural ectopic pregnancy associated with previous B-Lynch sutures

**DOI:** 10.1186/s12905-024-03027-w

**Published:** 2024-04-02

**Authors:** Junmiao Xiang, Fendang Chen, Zhuhua Cai, Ruru Bao

**Affiliations:** 1 Department of Gynecology, Ruian People’s Hospital, Wangsong Road, Ruian, 325000 Zhejiang China; 2 Department of Ultrasonography, Ruian People’s Hospital, Wangsong Road, Ruian, 325000 Zhejiang China

**Keywords:** Intramural ectopic pregnancy, Laparoscopy, B-Lynch sutures, Hysteroscopy, Intrauterine adhesions, Case report

## Abstract

**Background:**

Intramural ectopic pregnancy is a rare form of ectopic pregnancy that occurs within the myometrium. It is challenging to diagnose it early because of its nonspecific clinical presentation, and there is no consensus or guideline on the optimal management among gynecologists.

**Case presentation:**

We report a case of a 34-year-old woman who developed fundal intramural ectopic pregnancy after a previous caesarean section with B-Lynch suture. The B-Lynch suture was performed at 38 weeks of gestation for postpartum hemorrhage caused by refractory uterine atony about 8 years ago. Since then, the patient had oligomenorrhea. The diagnosis of intramural ectopic pregnancy was not confirmed by magnetic resonance imaging or ultrasound. An exploratory laparoscopy and hysteroscopy was performed to remove the gestational sac without significant bleeding. The surgery was successful and the patient recovered well. The patient was advised to monitor her β-HCG levels regularly until they returned to normal, and a follow-up pelvic ultrasound showed no complications. However, she has not been able to conceive or have an ectopic pregnancy so far.

**Conclusions:**

This case illustrates the difficulty of diagnosing intramural ectopic pregnancy, especially when it is associated with previous uterine surgery and B-Lynch suture. It also demonstrates the feasibility and safety of laparoscopic surgery for treating complete IUP, especially when the gestational sac is located close to the uterine serosa. However, the risk of uterine rupture and hemorrhage should be considered, and the patient should be informed of the possible complications and alternatives. Gynecologists should be familiar with various management strategies and customize the treatment plan according to the patient’s clinical situation and preferences.

## Introduction

Ectopic pregnancy is a condition in which the fertilized ovum implants outside the endometrial cavity, most commonly in the Fallopian tubes. Intramural ectopic pregnancy(IUP) is a rare form of ectopic pregnancy that occurs within the myometrium without any communication with the endometrial cavity, the Fallopian tubes, or the round ligament. Cesarean section, curettage and in vitro fertilization-embryo transfer are high-risk factors for IUP, which is a rare but potentially life-threatening condition. The population with these medical histories has increased in recent years, and many case reports of IUP have been published annually. However, there is a lack of relevant guidelines for IUP, so its etiology, pathogenesis, diagnosis and treatment need further analysis. We report a case of a complete fundal intramural ectopic pregnancy following previous B-Lynch sutures, which is a surgical technique to control postpartum hemorrhage. The diagnosis of IUP was challenging, as the imaging findings were inconclusive and could not differentiate between intramural or cornual pregnancy. The diagnosis was confirmed by hysteroscopy, which showed a gestational sac embedded in the uterine wall. The management of IUP is controversial, as there is no consensus or guideline on the optimal treatment among gynecologists. The choice of treatment depends on various factors, such as the patient’s preferences, the location and size of the gestational sac, the availability of resources, and the expertise of the surgeon.

## Case presentation

A 34-year-old woman, gravida 1, para 1, with 45 days of amenorrhea presented to our gynecological clinic with lower abdominal pain for 10 days, denying a history of vaginal bleeding. She underwent a lower segment caesarean section and B-Lynch uterine compression suture at 38 weeks for postpartum hemorrhage caused by refractory uterine atony about 8 years ago, oligomenorrhea occurred since then. Gynecological examination revealed a slightly enlarged uterus and mild tenderness on the right side of the fundus. The serum β-HCG concentration was 76947.29 m IU/ml.

**Fig. 1:**
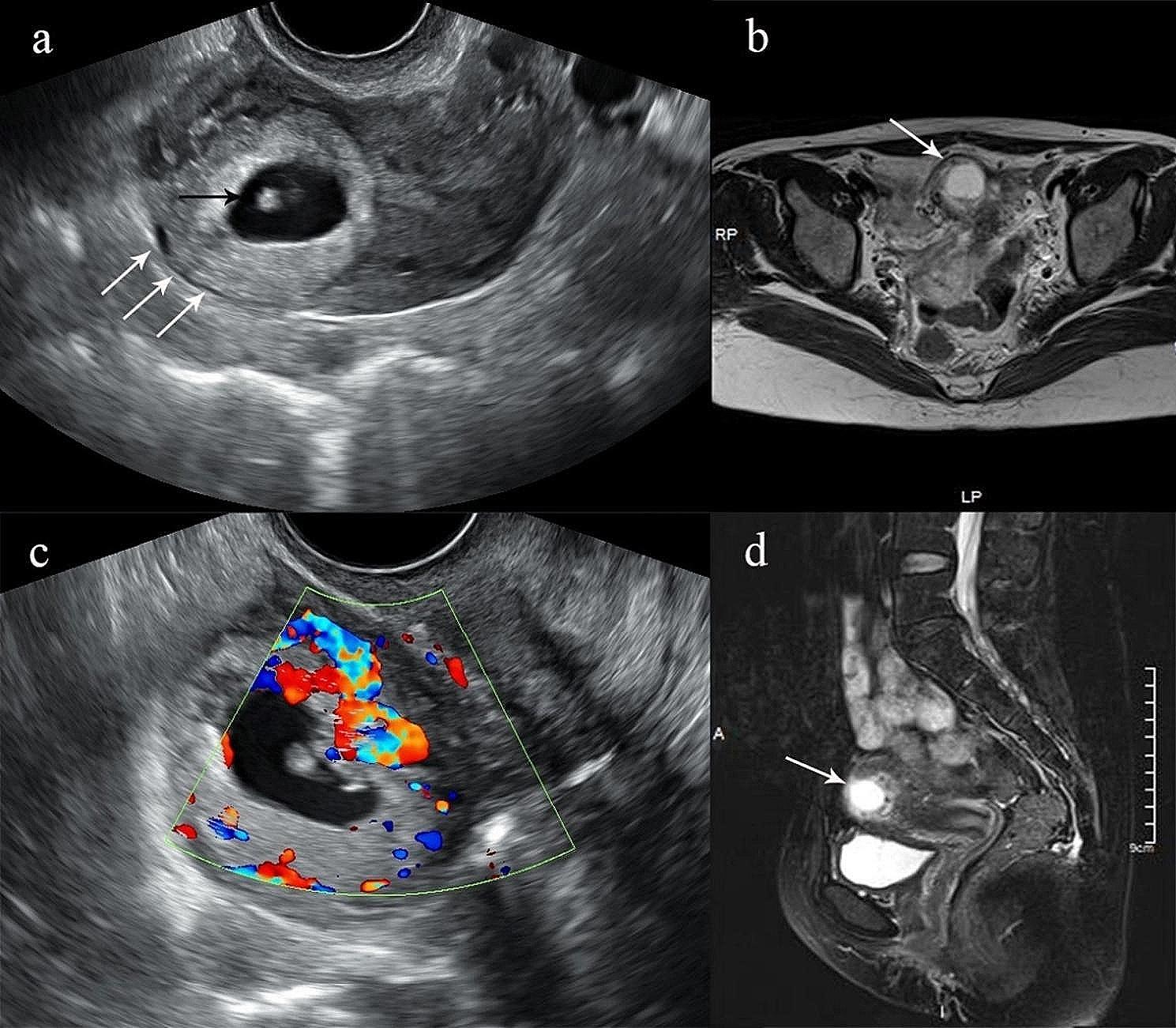
(a) Transvaginal ultrasound revealed a gestational sac (black arrow) embedded in the fundal myometrium near the right cornu of the uterus. The endometrial lining was indistinct and the uterine fundal serosa was thin, with a residual myometrial thickness of 2 mm (white arrow). (c) Color Doppler ultrasound demonstrated increased peritrophoblastic blood flow. (b) Magnetic resonance imaging (MRI) in the coronal plane demonstrated a gestational sac with intermediate to low echogenicity extending beyond the uterine contour, associated with marked myometrial thinning and indistinct demarcation from the uterine wall (white arrow). (d) MRI in the sagittal plane revealed an irregularly shaped uterus, with discontinuous endometrium and junctional zone, and a cystic lesion with high signal intensity at the uterine fundus (white arrow)

We performed a transvaginal ultrasound (TVS) scan, which showed an irregular uterus, with no endometrial lining. A gestational sac with a yolk sac and an embryonic pole was located in the right cornu, with a thin myometrium (2 mm) in the anterior wall of the fundus (Fig. 1a). Colour Doppler imaging revealed increased blood flow around the trophoblast (Fig. 1b). The sonographer misdiagnosed it as a right cornual pregnancy. Magnetic resonance imaging (MRI) confirmed a deformed uterus, with incomplete endometrium and junctional zone. A cystic structure at the fundus was not clearly separated from the myometrium, which was thinned adjacent to the cyst (Fig. 1d). The cyst protruded outward from the right side of the fundus, with a markedly thinned myometrium (Fig. 1c).

Because the diagnosis of right cornual pregnancy or IUP was unclear, exploratory laparoscopy and hysteroscopy were performed to further confirm this diagnosis.

Surgical Steps

- The surgery consisted of two main steps: laparoscopy and hysteroscopy.
- laparoscopy and hysteroscopy were performed to confirm the diagnosis of IUP and to remove the gestational sac from the myometrium.
- The surgical steps were as follows:

Step 1: hysteroscopy.

**Fig. 2:**
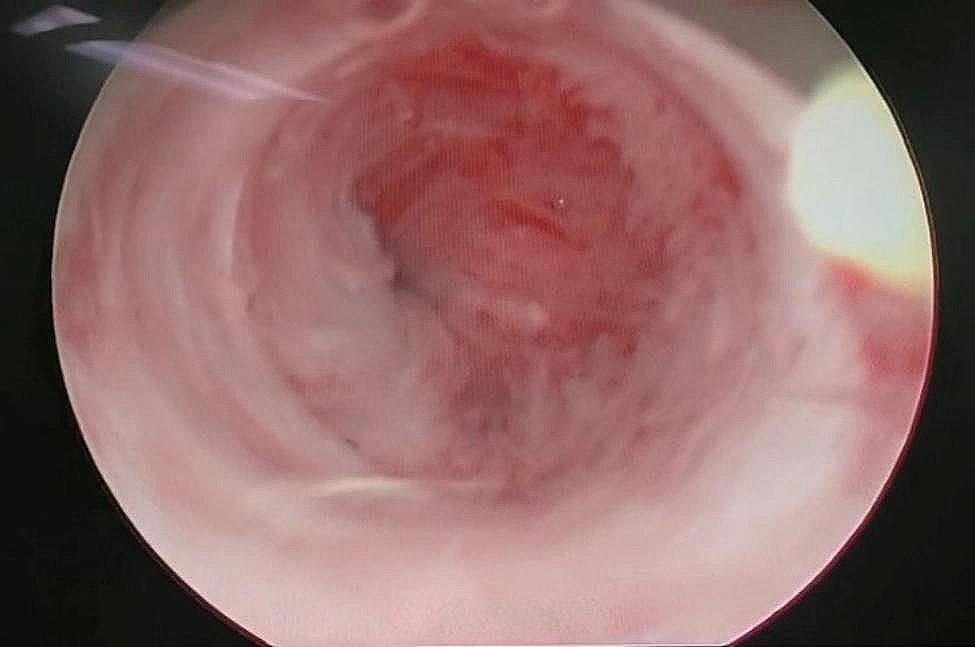
Under hysteroscopy, no evidence of a gestational sac was detected, and the uterine cavity was extremely narrow and fibrotic, with no endometrial lining

- Hysteroscopy was performed to examine the uterine cavity and the ostia.
- No sign of a gestational sac was found in the uterine cavity.
- The right ostium from the right fallopian tube was seen, but the left ostium was covered with an adhesive band.
- The uterine cavity was very narrow and fibrotic, and had no overlying endometrium, indicating severe intrauterine adhesions (Fig. 2).

Step 2: laparoscopy.

**Fig. 3:**
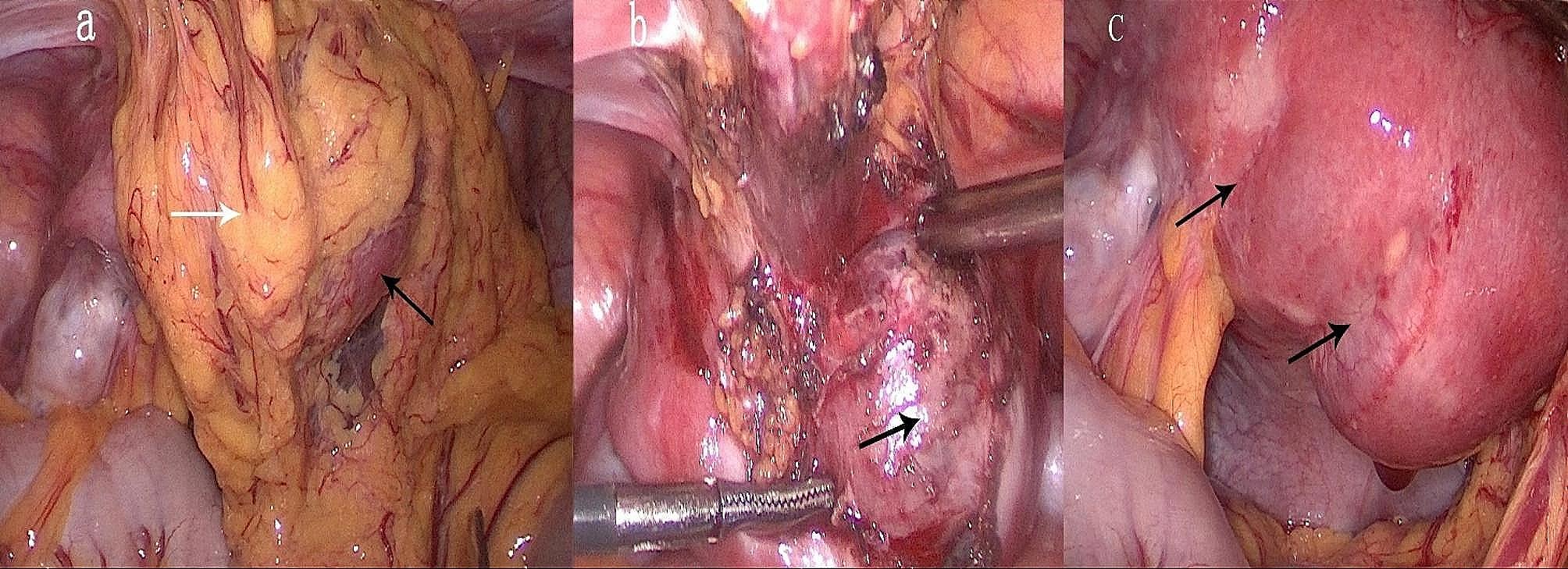
(a) Laparoscopic findings showed that the greater omentum (white arrow) was severely adhered to the anterior uterine wall and the fundus (black arrow). (b) After removing the adhesion, the gestational sac (black arrow) was found embedded in the myometrium and protruding from the right side of the fundus. (c) Two light blue longitudinal stripes with some vessels on their surface on the posterior uterine wall indicated the postoperative changes after B-Lynch sutures (black arrow)

- laparoscopy was performed to examine the uterus and the adnexa.
- The uterus was enlarged to the size of a 7-week gestation, with normal bilateral adnexa.
- The omentum and the entire anterior wall and fundus of the uterus were densely adherent (Fig. 3a).
- The posterior wall of the uterus showed two light blue longitudinal stripes with some vessels on the surface, indicating the postoperative changes after B-Lynch sutures (Fig. 3c).

Step 3: Removal of the adhesions and the gestational sac.

- The adhesions between the omentum and the uterus were removed by using electrocautery and scissors.
- A bluish-purple mass suggestive of the conception was found embedded in the myometrium and protruding from the right side of the fundus.
- This confirmed that the conception was not connected to the endometrial cavity.
- The serosa and myometrium above the gestational sac were thinned but not ruptured (Fig. 3b).

Step 4: Resection of the lesion and pathological examination.

- Diluted pituitrin hypophysin was injected into the myometrium around the mass to reduce bleeding.
- An incision was made over the bulging myometrium.
- The lesion was resected and sent for routine pathological examination, which confirmed the presence of chorionic villi and decidua.

Step 5: Suturing of the uterine defect.

- The uterine defect was sutured with 1 − 0 Vicryl in two layers: the endometrial and myometrial layers were sutured with interrupted stitches, and the seromyometrial layer was sutured with continuous locking stitches.

Surgical Outcomes and follow-up.

- The surgery was successful and the patient recovered well.
- The estimated blood loss during the operation was around 100 ml.
- The patient’s serum β-HCG level decreased to 8112.8 mIU/ml on the third postoperative day and was discharged in good condition.
- A follow-up pelvic ultrasound revealed no complications. The patient was instructed to monitor β-HCG levels regularly until they normalized, and to refrain from pregnancy for at least one year.
- Because of the desire for fertility, the patient underwent hysteroscopic adhesiolysis and estrogen replacement therapy in our hospital within one year after the surgery. The menstrual volume has increased compared to before, and the B-ultrasound showed that the endometrium was slightly thicker than before, but the patient still did not conceive or have an ectopic pregnancy so far.

## Discussion

IUP is considered as the rarest type of ectopic pregnancy, with a rate of less than 1% [1], while the rate of IUP is expected to increase in the future due to the growing number of caesarean deliveries. Here we report a case of IUP implanted into the fundal myometrium after caesarean section and B-Lynch uterine compression suture. The etiologies and pathogenesis of IUP are unknown, possible causes include caesarean section, in vitro fertilization/embryo transfer, myomectomy, adenomyosis, or pelvic infection [2]. B-Lynch suture is a surgical technique to control postpartum hemorrhage caused by uterine atony. It involves placing a compressive suture around the uterus to reduce its size and blood flow. In this case, B-Lynch suture was applied in a sling-like fashion on the right side of the fundus, which caused myometrial damage [3]. The blastocyst implanted into the myometrial defect through a microscopic dehiscent tract. Moreover, the mechanical effect and the interruption of uterine blood supply by B-Lynch suture resulted in intrauterine adhesions [4]. Because of the intrauterine adhesions and the absence of overlying endometrium, the fertilized ovum could not implant in the uterine cavity. In addition, the formation of the microscopic dehiscent tract in the myometrium made B-Lynch suture a risk factor for IUP. The diagnostic algorithm includes ultrasound, serum β-HCG levels, and a clinical examination. Early diagnosis of IUP can avoid serious complications. However, early diagnosis is sometimes difficult due to its various manifestations, and it might be mistaken for interstitial or cornual pregnancy. The diagnosis of IUP requires clear visualization of the endometrial–myometrial junction to delineate that the gestational sac is completely surrounded by the myometrium. TVS is generally the first imaging examination to be used [5], however, MRI may be a better way to evaluate the complete extent of this disease [6], and may help to delineate the uterine cavity better. In this case, the ultrasound scan showed that the gestational sac was located at the right side of the uterine fundus, and the myometrium near the lower endometrial cavity was thin, suggesting that the gestational sac was not completely surrounded by the uterine muscle. Therefore, the sonographer misdiagnosed it as a right cornual pregnancy. However, due to the endometrial fibrosis caused by B-Lynch, the MRI could not provide a full view of the endometrial–myometrial junction and endometrial lining, and thus could not distinguish the relationship between the gestational sac and the myometrium. So we did not recognize it as an IUP. It is worth mentioning that we hypothesized that there was a severe intrauterine adhesion because of the absence of endometrial lining and clinical symptoms of oligomenorrhea, until we proceeded with exploratory hysteroscopy to verify the conjecture. There is no universal treatment modality because of the few cases reported. Management of intramural pregnancies should be individualized. It depends on the location of the conception, degree of myometrial involvement, gestational age and desire to maintain pregnancy. Commonly available management options include expectant, medical (methotrexate ± vasopressin) and surgical (laparoscopy, hysteroscopy or laparotomy) treatment [7]. Women need to be informed that IUP can lead to massive bleeding and high maternal mortality rates if they continue with the pregnancy. Ramkrishna [8] pointed out that for patients with gestational age less than 8 weeks and no uterine rupture, medical treatment can be considered, but it has disadvantages such as long treatment duration, slow decline of serum β-HCG, and delayed surgical timing in case of uterine rupture. Surgical treatment includes laparoscopy, laparotomy, and hysteroscopy combined with laparoscopy [9], and the principle is to preserve the patient’s fertility function as much as possible. For cases where the gestational tissue is far from the serosal layer and difficult to locate by naked eye, hysteroscopy combined with TVS can accurately locate the implantation site of the gestational sac and clarify the relationship between the gestational sac and the endometrial cavity. In this case, considering that the IUP tissue has rich blood supply and the surrounding myometrium is thin, if conservative medical treatment is chosen, the risk of uterine rupture and massive bleeding is high. The patient has a strong desire for fertility, and surgical treatment can not only completely remove the lesion, shorten the treatment duration, but also repair the poorly healed scar after surgery. However, for patients with stable condition, small lesion, and clear diagnosis of IUP, ultrasound-guided puncture aspiration and local injection of MTX can be performed, which can preserve the integrity of the uterus and offer a new treatment option for IUP patients who have fertility requirements. LI et al [10]. first reported the use of uterine artery embolization and medication to treat IUP which reduced the risk of uterine rupture and massive bleeding, and provided technical support for preserving the fertility of the patients. B-Lynch suture is very successful in avoiding the need for hysterectomy during refractory postpartum hemorrhage that does not respond to medical treatment [11]. However, the impact of B-Lynch suture on future fertility potential needs further study. From this case, we learned that intrauterine adhesions are one of the important causes of IUP induced by B-Lynch, and also one of the difficulties in treating patients who have fertility requirements. In B-Lynch surgery, we should pay attention to reducing the damage to the uterine muscle layer and endometrium. If intrauterine adhesions occur after surgery, we need to remove the adhesions with hysteroscopy as soon as possible to restore the shape of the uterine cavity, and improve the endometrial growth with estrogen replacement therapy and reduce the impact on future fertility potential. After treatment, the β-hCG level should be closely monitored, and vaginal ultrasound and MRI should be performed if necessary, to be aware of the possibility of persistent ectopic pregnancy, or even more severe trophoblastic disease caused by IUP. We find this case quite unique for two reasons. One is that the gestational sac implanted into the microscopic dehiscent tract at the fundus caused by the B-Lynch uterine compression suture, rather than the caesarean section incision. The other is the fact that hysteroscopy revealed a severe intrauterine adhesion, which could be another reason for the result of IUP.

## Conclusion

Improved imaging examination machines could lead to more frequent detection of IUP in the future. It is important for gynecologists to be aware of the risk factors and maintain the suspicion of IUP in order to prevent potentially catastrophic consequences in its early stage. In this case, the radiologists and sonographers alerted the gynecologists that this case was not a normal intrauterine pregnancy, but the uterine adhesions prevented a definitive diagnosis by ultrasound and MRI. Therefore, a diagnostic hystero-laparoscopy not only confirmed the diagnosis, but also enabled early treatment, reducing the risk of uterine rupture and bleeding. Thus, multidisciplinary collaboration among gynecologists, radiologists, and sonographers is very important for early diagnosis and individualized management of complex IUP.

## Data Availability

The datasets used and/or analysed during the current study available from the corresponding author on reasonable request.

## Abbreviations

IUP: Intramural ectopic pregnancy
TVS: Transvaginal ultrasound
MRI: Magnetic Resonance Imaging
